# Not All Bacterial Outer-Membrane Proteins Are β-Barrels

**DOI:** 10.17912/micropub.biology.001394

**Published:** 2025-02-07

**Authors:** John Heido, Simon Keng, Hulya Poyrazoglu, Ekta Priyam, Sajith Jayasinghe

**Affiliations:** 1 Chemistry and Biochemistry, California State University San Marcos, San Marcos, California, United States

## Abstract

The discovery of Wza, an octomeric helical barrel integral bacterial outer-transmembrane protein, has challenged the widely held understanding that all integral outer-membrane proteins of Gram-negative bacteria are closed β-barrels composed of transmembrane β- strands. Wza is a member of the Outer-Membrane Polysaccharide Exporter family and our bioinformatics analysis suggests that other members of the family may also contain outer-membrane transmembrane segments that are helical. A review of the literature indicates that in addition to Wza, outer-membrane core complex proteins of the type IV secretion systems also contain transmembrane segments that are helical.

**Figure 1. Alpha Helical Transmembrane Segments in Bacterial Outer-Membrane Proteins:**
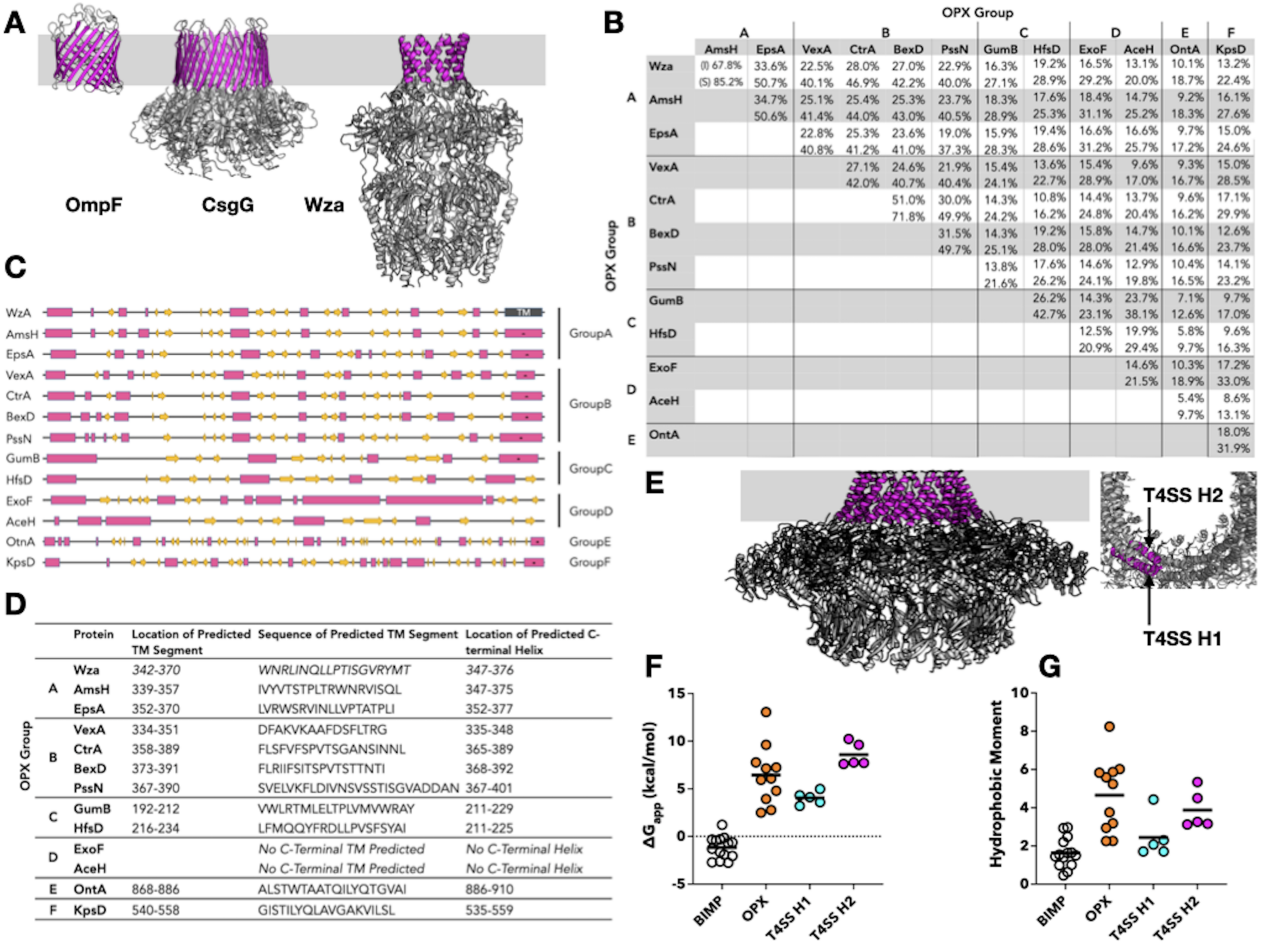
**(A)** Representative structures of three classes of bacterial outer-membrane proteins. Structure of the porin OmpF (PDB ID: 2OMF monomer) where the transmembrane (TM) region is composed of a 16- stranded β-barrel formed from a single polypeptide chain. Structure of the Curli secretion channel CsgG (PDB ID: 4UV3) where the TM region is composed of TM β-strands from nine individual polypeptide chains combining to form a 36-stranded β-barrel. Structure of the group 1 capsular polysaccharide transporter Wza (PDB ID: 2J58) where the TM region is composed of α-helical TM segments from eight polypeptide chains combining to form an α-helical barrel. **(B)** Sequence identity (top row in each entry) and similarity (bottom row in each entry) of group A, B, C, D, E, and F OPX proteins. Group A: Wza (UniProt-P0A930), AmsH (Q46629), EpsA (Q45407); Group B: BexD (P22236), CtrA (P0A0V9), PssN (Q27SU9), VexA Q04976); Group C: GumB (Q456768), HfsD (Q(A5L5); Group D: ExoF (Q02728), AceH (Q8RR80); Group E: OntA (Q56653); Group F: KpsD (Q03961). OPX proteins from groups A, B, C, and D show high sequence similarity to each other (ranging from 40 to 85% ) and exhibit more than 25 % sequence similarity to Wza. Group E and F proteins are much larger than other OPX proteins and exhibit less similarity to Wza. **(C)** Predicted secondary structure distribution of thirteen OPX proteins representing the six OPX classes. β-strands are shown as yellow arrows while helices are shown as pink rectangles. Secondary structure predicted using PsiPred. All proteins except ExoF and AceH from group D indicate the presence of a C-terminal segment (*). MPEx and PsiPred also predict these regions to be possible TM segments. **(D)** C-terminal helical TM segments predicted in representative examples of OPX proteins. The transmembrane segment of Wza, the only OPX protein whose structure is known, is located at the C-terminus of the protein. We used PsiPred (MEMSAT SVM) and MPEx to identify any possible C-terminal TM segments present in OPX proteins. We also predicted the secondary structure distribution of these proteins using PsiPred and identified the most C-terminal helix. All of the proteins investigated, except the two from group D, contain a C-terminal helical region and these are predicted to be possible TM regions. **(E)** Structure of the outer-membrane core complex of T4SS of *Xanthomonas citri* (PDB ID 6GYB). Side (left) and top (right) view of the complex composed of VirB9 and VirB10 are shown in ribbon representation. The outer-membrane α-helical transmembrane segments of VirB10 are shown in magenta. The TM helices form two concentric rings that line the pore of the T4SS outer-membrane channel. VirB10 appears to be present in nearly all T4SS complexes (Sheedlo 2022) and therefore, the presence of helical outer-membrane segments may be a common feature of T4SSs. **(F)** Inner membrane insertion potential (ΔG _app_ ) of helical TM segments of outer-membrane proteins. Almost all of the TM segments bitopic inner membrane proteins (BIMP) inserted into the bacterial inner membrane (by the translocon) exhibit ΔG _app_ <0 (left, black circles) while the ΔG _app_ values of the α-helical TM segments of outer-membrane OPX (orange circles) and T4SS proteins (blue for helix 1 and pink, for helix 2, circles) have ΔG _app_ >0 suggesting that the helical TM segments from the outer-membrane proteins investigated here have low inner membrane insertion potential. **(G)** Hydrophobic moment (amphipathicity) of helical TM segments of outer-membrane proteins. Helical TM segments from the OPX proteins and TM helix 2 of the T4SSs have hydrophobic moments that are on average larger than those observed for the helical TM segments of bitopic inner membrane proteins. It has been suggested that the amphipathic nature of the helical TM segments facilitate membrane insertion (Dunstand 2015, Jeeves 2015). In **F** and **G** the bars represent the means of each dataset.

## Description

Almost all integral membrane proteins found in the outer-membrane of Gram-negative bacteria adopt a β-barrel architecture (Kleinschmidt, 2015, Fairman et al., 2011) . These outer-membrane proteins (OMPs) contain anti-parallel transmembrane (TM) beta-strands that are amphipathic with the non-polar face interacting with the hydrophobic region of the lipid membrane. Typically, the TM β-strands of OMPs, as exemplified by OmpA (Koebnik et al., 2000) , are segments of a single polypeptide chain with the N- and C-terminal stands interacting to form a closed barrel ( Figure 1A ). There is also an emerging class of β-barrel OMPs, represented by the Curli secretion channel CsgG (Cao et al., 2014) , where the β-barrel is formed from TM β-strands contributed from individual subunits of a homo multimer ( Figure 1A ). It has long been thought that the outer-membrane of Gram-negative bacteria contains only β-barrel integral membrane proteins. This concept changed in 2006 with the discovery of Wza, a 359-residue integral OMP involved in the transport of capsular polysaccharides (Dong et al., 2006) . Based on the crystal structure, Dong et. al. proposed that Wza contains an α-helical C-terminal transmembrane segment spanning residues 345 to 376 ( Figure 1A ). In the functional assembly the helical TM segments of eight Wza monomers associate to form an α-helical barrel structure that forms a pore in the outer-membrane. The discovery of Wza suggests that helical TM segments may be a more prevalent feature of Gram-negative bacterial OMPs. A review of the literature and a bioinformatics analysis was carried out to identify other OMPs with helical TM segments.

***A C-terminal helical outer-membrane TM segment may be common in OPX proteins:*** Integral membrane proteins of the bacterial outer-membrane are typically β-barrel proteins containing β-strand TM segments. Until recently the one exception to this observation was the outer-membrane protein Wza which contains a C-terminal helical TM segment. Wza belongs to the Outer-Membrane Polysaccharide Exporter (OPX), also known as the Outer-Membrane Auxiliary (OMA), family of proteins (Cuthbertson et al., 2009, Sande et al., 2019) . The OPX family of proteins are the final components in the transport of capsular and extra-cellular polysaccharides across the bacterial outer-membrane and are further subdivided into six groups (A through F) (Cuthbertson et al., 2009) . Wza is a group A OPX and is involved in the transport of group 1 capsular polysaccharides. Although not all OPX protein appear to contain an outer-membrane spanning domain (Saïdi et al., 2022), it is possible that those that do are anchored to the outer-membrane via a helical TM segment. A sequence analysis was undertaken to determine if other OPX proteins might contain a C-terminal helix capable of anchoring these proteins to the bacterial outer-membrane.

Thirteen protein sequences representing each of the OPX groups were compared to identify similarities that would support the hypothesis that other OPX proteins may have a structural organization similar to Wza. Pairwise sequence alignments of the thirteen OPX proteins indicates similarities ranging from 9 to 85% between the different members of the family ( Figure 1B ). High sequence similarities (ranging from 40 to 85% ) are observed for proteins within groups A, B, and C. Additionally, almost all members from OPX groups A, B, C, and D exhibit more than 25 % sequence similarity to Wza. Proteins belonging to groups E and F, which are much larger than the other OPX proteins, show less (~ 18-22%) sequence similarity to Wza. The sequence similarly between Wza and the OPX proteins from groups A, B, C, and D suggest the possibility that proteins from these four groups could have a membrane architecture similar to that of Wza. This possibility is supported by the observation that all except two proteins (those from group D) have very similar secondary structure distributions to Wza ( Figure 1C and also (Cuthbertson et al., 2009) . The outer-membrane helical TM segment of Wza is found at the extreme C-terminal region spanning residues 345 - 376 (Dong et al., 2006) . All of the group A, B, C, E and F proteins investigated here, but not the two from group D, contain a C-terminal helical region which could be embedded in the OM ( Figure 1D ). Analysis of the protein sequences using MPEx (Snider et al., 2009) and PsiPred (Jones, 1999, Buchan et al., 2013) identified putative C-terminal TM regions, in all proteins investigated except the two representatives from group D ( Figure 1D ). These predicted TM segments correspond to the C-terminal helical regions identified using secondary structure analysis. Taken together these observations point to the distinct possibility that at least group A, B, C, E and F OPX family members may also have an α-helical transmembrane segment which helps to form a pore in the bacterial outer-membrane.

***A helical TM segment is also found in the outer-membrane pore component of type IV section systems:*** In addition to Wza, at least six other proteins, all outer-membrane core complex components of the type IV secretion system (T4SS), have been identified as containing helical TM segments. T4SSs are a diverse family of bacterial protein complexes that mediate the transfer of DNA and protein components to target cells (Christie et al., 2014, Grohmann et al., 2018, Christie, 2019, Sgro et al., 2019, Sheedlo et al., 2022). The canonical T4SS complex from *Agrobacterium tumefaciens* is composed of 12 proteins, named VirB1 to 11 and VirD4, that spans the inner-membrane, the periplasm and the outer-membrane. The outer-membrane core is composed of three proteins, VirB7, VirB9, and VirB10, of which VirB10 forms an outer-membrane pore (Sgro et al., 2019) . In the case of VirB10 from *Xanthomonas citri (* Sgro et al., 2018), the outer-membrane pore is composed of two concentric rings of C-terminal TM helices from 14 VirB10 molecules, where the α-helices of the outer-ring contact the membrane while the helices of the inner ring line the pore ( Figure 1E ). A similar architecture of TM helices is observed in VirB10 equivalents from the outer-membrane core complexes of at least five other type IV secretion systems: *E. coli* TraF, a VirB10 homolog encoded by the pKM101 plasmid (Chandran et al., 2009) , *E. coli* TrwE, a VirB10 homolog encoded by the R388 plasmid (Macé et al., 2022) , *Salmonella* TraB, a VirB10 homolog encoded by the F plasmid (Liu et al., 2022, Amin et al., 2021) , CagY, the VirB10 homolog from *Helicobacter pylori (* Chung et al., 2019), and DotG, the VirB10 homolog from *Legionella pneumpphila* (Sheedlo et al., 2021) . Despite the presence of a wide variety of T4SSs, VirB10 appears to be a core component of the assembly, and the C-terminus of these proteins appears to be conserved (Sheedlo et al., 2022) . Thus, the presence of C-terminal helical TM segments may be a common feature of T4SSs.

***Helical TM segments of outer-membrane proteins are in general less hydrophobic and more amphipathic than helical TM segments of inner-membrane proteins:*** Membrane proteins destined for the bacterial outer-membrane must first pass through the inner membrane. One route through the inner membrane involves the SecYEG translocon which helps the transport of proteins and is also responsible for facilitating the insertion of proteins into the inner membrane. The insertion of TM segments into the inner membrane by SecYEG appears to involve partitioning of protein segments between the translocon channel and the lipid membrane, and it is thought hydrophobicity plays a role in the identification of segments destined to become inner membrane TMs (Hessa et al., 2007, Cymer et al., 2015, Schow et al., 2011, Öjemalm et al., 2013). If the helical TM segments of outer-membrane proteins traverse through the SecYEG translocon their hydrophobicity must be sufficiently low to avoid accidental insertion into the inner membrane (IM).

The potential for a given amino acid sequence to be recognized as a TM and be inserted into the IM, the membrane insertion potential (ΔG _app_ ) can be estimated (Hessa et al., 2005, 2007, Öjemalm et al., 2013). Based on the observation that while TM segments of bitopic (single spanning) integral membrane proteins almost always exhibit ΔG _app_ < 0 kcal/mol and secreted proteins almost never contain segments with ΔG _app_ < 0 (Hessa et al., 2007) , it is generally taken that an amino acid segment with a negative ΔG _app_ will be recognized by the translocon to be TM and be inserted in to the inner-membrane. We compared ΔG _app_ values of the α-helical TM segments of outer-membrane OPX and Type IV secretion system proteins to values from helical TM segments of a collection of bitopic integral inner membrane proteins. All of the helical TM segments from the outer-membrane proteins exhibit ΔG _app_ >0, whereas almost all of the TM segments from bitopic bacterial inner membrane proteins exhibit ΔG _app_ < 0, suggesting that the helical TM segments from outer-membrane proteins investigated here have low inner membrane insertion potential ( Figure 1F ). This observation supports our expectation that low membrane insertion potential, or hydrophobicity, allows outer-membrane helical TM segments to pass through the translocon without accidentally being inserted into the inner membrane.

It should be noted that a positive ΔG _app_ value does not by itself preclude insertion into the inner membrane. In fact a significant number of TM segments from polytopic integral membrane proteins have been found to have quite positive ΔG _app_ values, to the extent that some segments are not even initially recognized as TM by the translocon (Marothy & Elofsson, 2015, Hedin et al., 2010, Whitley et al., 2021). The insertion of TM segments with positive ΔG _app_ appears to depend on surrounding sequence features such as the presence of flanking charged amino acids and the presence of TM segments with high insertion potential (Marothy & Elofsson, 2015, Hedin et al., 2010). Such features are lacking in the outer-membrane proteins discussed here presumably ensuring that the TM segments of these proteins pass through the translocon.

***Outer-Membrane helical TM segments have large hydrophobic moments that may aid in their insertion into the outer-membrane:*** Once proteins destined for the outer-membrane pass through the translocon they are transported through the periplasm and targeted to the bacterial outer-membrane at which point the TM segments insert into the outer-membrane. In the case of integral beta-barrel proteins, which comprise the majority of outer-membrane proteins, the details of this process are established and integration into the outer-membrane involves the BAM complex (Tommassen, 2010, Kim et al., 2012, Tomasek & Kahne, 2021) . Although mechanistic details of the integration of outer-membrane proteins with helical TM segments is not known, at least in the case of Wza the process does not appear to involve the BAM complex (Dunstan et al., 2015) . It has been proposed that integration of outer-membrane proteins that do not contain a traditional β-barrel architecture follows a novel pathway independent of BAM (Dunstan et al., 2015) and that perhaps the amphipathic TM regions of these proteins facilitate membrane insertion (Dunstan et al., 2015, Jeeves & Knowles, 2015) . The TM segments of OPX and T4SS proteins are amphipathic ( Figure 1G ), especially those from the OPX proteins and TM helix 2 of the T4SSs, which have hydrophobic moments that are on average larger than those found in bitopic inner membrane protein TM segments. The helical TM segments of the OPX and T4SS proteins form membrane channels and we expect their hydrophobic and hydrophilic amino acids to form membrane interacting and pore lining regions respectively resulting in a large hydrophobic moment.

**Conclusion:** Although most proteins embedded in the bacterial outer-membrane adopt a β-barrel architecture members of at least two classes of proteins appear to contain helical TM segments embedded in the outer-membrane. The identification of these proteins changes the long-held understanding that all bacterial outer-membrane proteins were β-barrels and opens up the possibility that other classes of outer-membrane proteins may also contain TM helices.

## Methods

***Selection of Membrane Proteins and TM segments:*** Fifteen bitopic (single spanning) bacterial inner membrane proteins were identified using the Membranome 3.0 database (Lomize, Hage, et al., 2017, Lomize, et al., 2017) and the TM segments of these proteins were identified in consultation with published accounts.

***Prediction of Helical Segments with Possible TM Segments:*** MPEx (Snider et al., 2009) and PsiPred (MEMSAT SVM) (Buchan et al., 2013, Jones, 1999) were used to predict the presence of transmembrane segments. In the case of MPEx, predictions were carried out using the Wimley-White octanol scale (Wimley & White, 1996) with a window size of 19 residues (defaults). PsiPred was used to predict the secondary structure distribution of each protein from which the helical secondary structure regions were identified.

***Determining Hydrophobicity and Amphipathicity of TM segments:*** Hydrophobicity, as qualified by the membrane insertion potential, ΔG _app_ , of each TM segment was calculated using the ΔG prediction server at (https://dgpred.cbr.su.se/index.php?p=home, (Hessa et al., 2007)). Amphipathicity, the asymmetric distribution of non-polar and polar amino acids, was quantified using the hydrophobic moment which was calculated using the translocon scale in the totalizer module of MPEx (Snider et al., 2009) . When the transmembrane domain of a protein was formed from the association of more than one monomer, calculations were performed on a helix from a single monomer and the TM segment was defined as indicated in the individual primary reports.
