# Corrigendum: Not All Bacterial Outer-Membrane Proteins Are β-Barrels

**DOI:** 10.17912/micropub.biology.001556

**Published:** 2025-02-24

## Description

Two authors, **Pream Kadevari and Bin Tu, were inadvertently left off the author list in the original submission. These authors have now been added.**

